# Supplementary material for: Dimer Rhenium Tetrafluoride with a Triple Bond Re-Re: Structure, Bond Strength
Source: Molecules. 2023 Apr 23;28(9):3665. doi: 10.3390/molecules28093665 (PMC10179812; doi:10.3390/molecules28093665)
Supplement: Supplementary file 1 [file molecules-28-03665-s001.zip › molecules-2341568-supplementary.pdf]

SUPPLEMENTARY MATERIALS

Dimer Rhenium Tetrafluoride with a Triple Bond Re-Re: Structure, Bond Strength

Nina I. Giricheva [1], Natalia V. Tverdova [2], Valery V. Sliznev [2] and Georgiy V. Girichev [2,*]

[1] Nanomaterial Research Institute, Ivanovo State University, Ermak Str. 39, 153025 Ivanovo, Russia; n.i.giricheva@mail.ru
[2] Department of Physics, Ivanovo State University of Chemistry and Technology, Sheremetevsky Ave. 7, 153000 Ivanovo, Russia; tverdova@isuct.ru (N.V.T.); sliznev@mail.ru (V.V.S.)
* Correspondence: g.v.girichev@mail.ru; Tel.: +7-4932-35-98-74

Table S1. Disagreement factor R_f and calculated vibration amplitude *l*(F5…F10) versus the torsion vibration frequency of the Re_2F_8 molecule.

ν_{tors}	20 см$^{-1}$	30 см$^{-1}$		40 см$^{-1}$	50 см$^{-1}$
R_f, %	5.061	4.832		5.145	5.491
	l_{calc}[1]	l_{calc}	l_{exp}	l_{calc}	l_{calc}
F5…F10, Å	0.432	0.314	0.315(15)	0.260	0.231

[1] Calculated (using different values of the torsion force constant and frequency) and experimental vibration amplitudes of the Re_2F_8 molecule.

Table S2. The calculated and experimental structural characteristics of the $[Re_2X_8]^{2-}$ (X=F, Cl, Br).

		r(Re-Re), Å	r(Re-X), Å	α(XReRe),°	ν(Re-Re), cm^{-1}	ν_{tors} cm^{-1}	ν(Re-X) [a] cm^{-1}
$[Re_2F_8]^{2-}$	calc	2.231	1.977	104.2	337	66	485 - 579
	exp [15]	2.188(3)	1.92 - 1.96(3)	99.9 - 102.6(4)	317		525 - 568
$[Re_2Cl_8]^{2-}$	calc	2.216	2.357	104.5	292	44	267 - 336
	exp [13]	2.224(1)	2.330(2)	103.4(5)	274 [12]		361 [12]
$[Re_2Br_8]^{2-}$	calc	2.225	2.512	105.2	287	25	159 - 224
	exp [13]	2.226(4)	2.467(4)	104.6(4)	276 [12]		211 [12]

[a] the range of eight stretching Re-X vibration frequencies.

Table S3. Conditions of the synchronous GED/MS experiments.

Nozzle-to plate distances, mm	**338**	**598**
Number of recorded plates	7	7
Electron beam current, μA	2.7	0.6
Wavelength of electrons, Å	0.04329(8)	0.04335(8)
Temperature of the effusion cell, K	471(5)	471(5)
Average exposure time, s	180	105
Residual gas pressure in diffraction chamber, Torr	$1.3 \cdot 10^{-6}$	$3.1 \cdot 10^{-6}$
s-range/Δs, Å^{-1}	2.4-23.3/0.1	1.1-15.0/0.1
Ionization voltage, V	50	50

Table S4. The vibrational frequency values of the molecule Re_2F_8 obtained in three variants DFT.

Frequency, cm^{-1}	PBE0/qRECP-1	B3LYP/qRECP-2	PBE/RECP-3
$\omega_1(a_{1g})$	749	710	704
$\omega_2(e_u)$	711	687	677
$\omega_3(a_{2u})$	699	672	665
$\omega_4(e_g)$	689	668	657
$\omega_5(b_{1g})$	670	642	638
$\omega_6(b_{2u})$	617	594	597
$\omega_7(a_{1g})$	370	345	338
$\omega_8(b_{2g})$	325	309	312
$\omega_9(b_{1u})$	312	296	304
$\omega_{10}(a_{2u})$	272	271	265
$\omega_{11}(e_g)$	221	207	217
$\omega_{12}(a_{1g})$	193	198	185
$\omega_{13}(e_u)$	213	197	207
$\omega_{14}(e_g)$	193	186	186
$\omega_{15}(b_{1g})$	170	183	161
$\omega_{16}(b_{2u})$	172	177	173
$\omega_{17}(e_u)$	131	147	125
$\omega_{18}(a_{1u})$	40	10	43

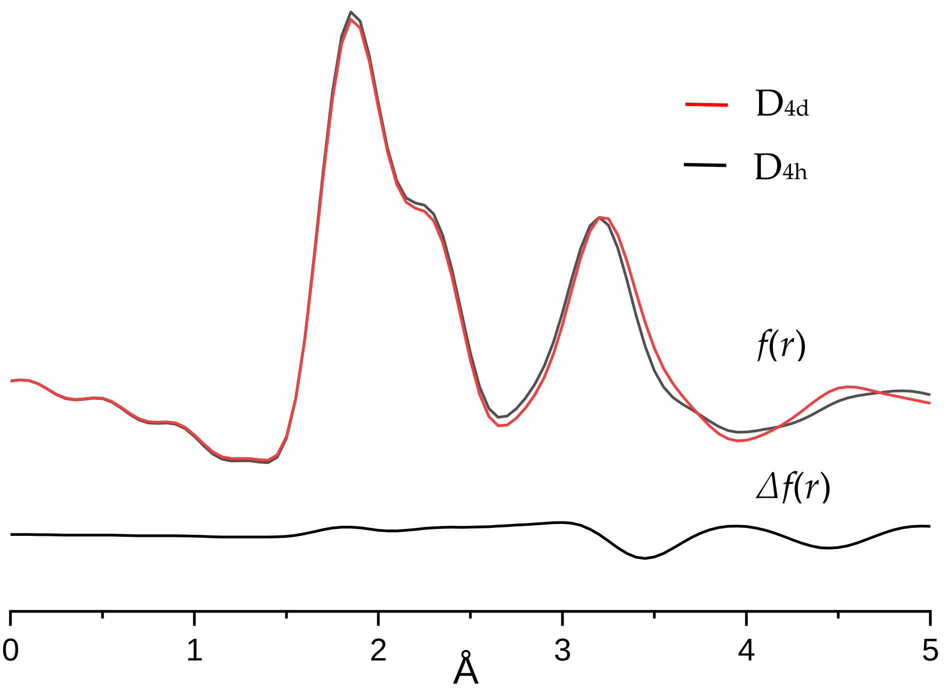

Figure S1. Theoretical functions f(r) for models D_{4h} and D_{4d} of the Re_2F_8 molecule and difference curve Δf(r).

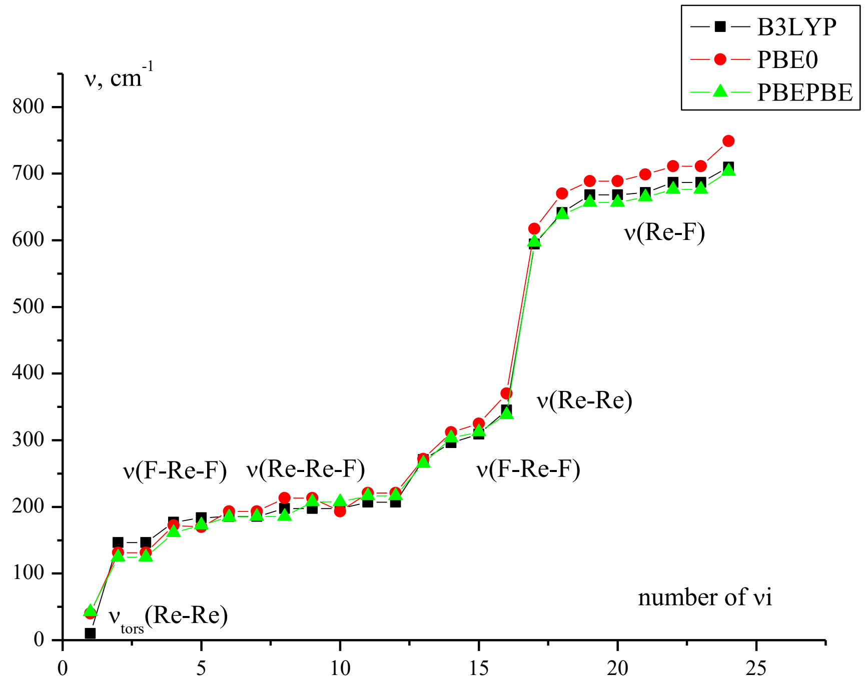

Figure S2. The values of vibrational frequencies of the molecule Re_2F_8 obtained in three variants DFT.
